# Stereotactic MR-Guided Radiotherapy for Pancreatic Tumors: Dosimetric Benefit of Adaptation and First Clinical Results in a Prospective Registry Study

**DOI:** 10.3389/fonc.2022.842402

**Published:** 2022-03-09

**Authors:** Morgan Michalet, Karl Bordeau, Marie Cantaloube, Simon Valdenaire, Pierre Debuire, Sebastien Simeon, Fabienne Portales, Roxana Draghici, Marc Ychou, Eric Assenat, Marie Dupuy, Sophie Gourgou, Pierre-Emmanuel Colombo, Sebastien Carrere, François-Regis Souche, Norbert Aillères, Pascal Fenoglietto, David Azria, Olivier Riou

**Affiliations:** ^1^ University Federation of Radiation Oncology of Mediterranean Occitanie, Montpellier Cancer Institute (ICM), Univ Montpellier, INSERM U1194 Institut de Recherche en Cancérologie de Montpellier (IRCM), Montpellier, France; ^2^ Medical Oncology Department, Institut du Cancer de Montpellier (ICM), Montpellier Cancer Institute, Univ Montpellier, Montpellier, France; ^3^ Medical Oncology Department, Centre Hospitalier Universitaire (CHU) St Eloi, Montpellier, France; ^4^ Biometrics Unit Institut du Cancer de Montpellier (ICM), Montpellier Cancer Institute, Univ Montpellier, Montpellier, France; ^5^ Digestive Surgery Department, Institut du Cancer de Montpellier (ICM), Montpellier Cancer Institute, Univ Montpellier, Montpellier, France; ^6^ Surgical Department, Centre Hospitalier Universitaire (CHU) St Eloi, Montpellier, France

**Keywords:** stereotactic MR-guided adaptive radiotherapy, stereotactic body radiation therapy, pancreatic cancer, pancreatic tumors, locally advanced pancreatic cancer, borderline resectable pancreatic cancers, adaptive radiotherapy, image guided radiotherapy (IGRT)

## Abstract

**Introduction:**

Stereotactic MR-guided adaptive radiotherapy (SMART) is an attractive modality of radiotherapy for pancreatic tumors. The objectives of this prospective registry study were to report the dosimetric benefits of daily adaptation of SMART and the first clinical results in pancreatic tumors.

**Materials and Methods:**

All patients treated in our center with SMART for a pancreatic tumor were included. Patients were planned for five daily-adapted fractions on consecutive days. Endpoints were acute toxicities, late toxicities, impact of adaptive treatment on target volume coverage and organs at risk (OAR) sparing, local control (LC) rate, distant metastasis-free survival (DMFS), and overall survival (OS).

**Results:**

Thirty consecutive patients were included between October 2019 and April 2021. The median dose prescription was 50 Gy. No patient presented grade > 2 acute toxicities. The most frequent grade 1–2 toxicities were asthenia (40%), abdominal pain (40%), and nausea (43%). Daily adaptation significantly improved planning target volume (PTV) and gross tumor volume (GTV) coverage and OAR sparing. With a median follow-up of 9.7 months, the median OS, 6-month OS, and 1-year OS were 14.1 months, 89% (95% CI: 70%–96%), and 75% (95% CI: 51%–88%), respectively, from SMART completion. LC at 6 months and 1 year was respectively 97% (95% CI: 79–99.5%) and 86% (95% CI: 61%–95%). There were no grade > 2 late toxicities. With a median follow-up of 10.64 months, locally advanced pancreatic cancer (LAPC) and borderline resectable pancreatic cancer (BRPC) patients (22 patients) had a median OS, 6-month OS, and 1-year OS from SMART completion of 14.1 months, 76% (95% CI: 51%–89%), and 70% (95% CI: 45%–85%), respectively. Nine patients underwent surgical resection (42.1% of patients with initial LAPC and 33.3% of patients with BRPC), with negative margins (R0). Resected patients had a significantly better OS as compared to unresected patients (p = 0.0219, hazard ratio (HR) = 5.78 (95% CI: 1.29–25.9)).

**Conclusion:**

SMART for pancreatic tumors is feasible without limiting toxicities. Daily adaptation demonstrated a benefit for tumor coverage and OAR sparing. The severity of observed acute and late toxicities was low. OS and LC rates were promising. SMART achieved a high secondary resection rate in LAPC patients. Surgery after SMART seemed to be feasible and might increase OS in these patients.

## Introduction

Pancreatic adenocarcinoma (PA) is the 10th cause of cancer in Europe and the United States and the 4th cause of cancer mortality. The 5-year overall survival (OS) is 9% for all stages, mainly due to a frequent metastatic spread (1). Surgical resection is the only curative modality, but only 10% of these cancers are resectable at diagnosis. On the other hand, 30% are considered unresectable or locally advanced (2). In locally advanced pancreatic cancer (LAPC), chemoradiotherapy is a frequent option after induction chemotherapy, since the phase III trial GERCOR LAP 07 demonstrated a benefit in terms of local control (LC) and delayed chemotherapy reintroduction as compared to chemotherapy (gemcitabine) alone, despite no advantage in terms of OS (3). These results were later confirmed by other studies on chemoradiotherapy, as suggested by a meta-analysis (4).

Stereotactic body radiotherapy (SBRT) is an attractive modality of radiotherapy in this indication for three main reasons: 1) possibility to deliver higher biologically equivalent doses (BED) in these radioresistant tumors, 2) modality allowing better organ at risk (OAR) sparing, and 3) decreased number of fractions in these patients with limited life expectancy with consequential improved quality of life. Recent data suggest an excellent LC with this treatment modality (5, 6), but the proximity of OARs limits the use of this technique.

Stereotactic MR-guided adaptive radiotherapy (SMART) is a technique combining X-ray beam delivery, daily adaptive treatment planning, and gating/tracking possibility through continuous cine-MR images (7, 8). MRIdian Linac^®^ is a radiotherapy device developed by ViewRay, coupling a 0.35-tesla MR-imaging system with a multileaf collimator-equipped linear accelerator (9). It is particularly adapted to pancreatic SBRT, improving the delineation accuracy thanks to better soft tissue MR contrast as compared to CT scan, sparing OARs by adaptation of treatment to the daily anatomy, and tracking the target with cine-MRI during irradiation. A retrospective study suggested an increase of OS with dose-escalated SMART in unresectable pancreatic cancers (10).

The objective of this study was to report the dosimetric benefits of daily adaptation of SMART and the first clinical results in pancreatic tumors.

## Methods and Materials

### Patient Selection

All patients treated with SMART for a pancreatic tumor at the Montpellier Cancer Institute from October 2019 to April 2021 were included.

Patients with non-metastatic unresectable pancreatic adenocarcinoma were first treated with induction chemotherapy and had stable or responsive disease. Metastatic pancreatic adenocarcinoma patients could be included in the study in case of metastatic complete or near-complete response to chemotherapy with a residual primary tumor. Primary tumors other than adenocarcinoma and metastatic lesions to the pancreas from other primaries could be included in the study. The indication of SMART had to be validated in a multidisciplinary tumor board. Histological confirmation was required. Other inclusion criteria were Eastern Cooperative Oncology Group (ECOG) performance status = 0 or 1, no previous abdominal radiotherapy, no MRI contraindication (presence of non-MRI compatible implanted cardiac devices, claustrophobia, psychiatric disorders, and metal objects), and no duodenal invasion on endoscopy.

This study was registered in the Health Data Hub (registration number: #1802) and was approved by our local research committee (2020/01). All patients signed an informed consent form before treatment.

### Simulation

All patients underwent CT simulation directly followed by 0.35-T MRI simulation using the MRIdian^®^ apparatus to ensure reproducibility of the anatomic configuration. MR and CT images were rigidly registered for target volume delineation, while only the MR images were used for OAR delineation. A 1.5-T MRI simulation in our radiology department was also required to allow better tumor visualization and improve gross tumor volume (GTV) delineation after registration with MRIdian^®^ images. Patients in all simulation exams were injected with contrast agents unless contraindication. Patients were asked to fast for at least 3 to 4 h prior to all simulation exams (and every fraction). Patients were in a supine position with arms down at their sides, and immobilization was obtained with a Totim^®^ device. Furthermore, for dose calculation, CT to MR image registration was performed using an elastic registration algorithm. During the CT simulation, MRI dummy surface coils with similar electron attenuation properties to real MRI coils were placed on the custom immobilization device. MR images were acquired with true fast imaging with steady-state free precession (TRUFISP) sequences (T1/T2 weighted, breath-hold technique (physiologic end-expiration), 17 to 25 s, 1.6 × 1.6 × 3 mm or 1.5 × 1.5 × 3 mm resolution, 45 × 45 × 24 to 54 × 47 × 43 maximum field of view).

### Breath-Hold Procedure

All patients were simulated and treated with a breath-hold technique. All the patients benefited after the first medical consultation from a respiratory coaching session by a radiotherapy nurse. They received a document explaining the respiratory breath-hold procedure and the terms that were going to be used during simulation and treatment. Patients were asked to perform respiratory breath-hold exercises at home. Another respiratory coaching session was performed directly before the first simulation. Breath-hold was achieved by voice guidance by the radiotherapy technicians at simulation and treatment. No abdominal compression was used. No specific visual coaching system was used. The quality and reproducibility of breath-hold were checked by continuous cine-MR guidance during simulation and treatment. Breath-hold was performed in physiologic end-expiration.

### Treatment Planning

The tumoral GTV (GTV T) was delineated using the data from CT and MRI. Suspect regional lymph nodes were also delineated if required (nodal GTV (GTV N)). An isotropic margin of 3 mm was used for the planning target volume (PTV) extension. OAR was delineated on MRIdian^®^ simulation images. OAR dose constraints are listed in **Table 1**. Priority was given to OAR dose constraints. An optimization structure (PTV optimized or PTVopt) was created as follows: PTVopt = PTV − (digestive OAR + 5 mm). The median prescribed dose was 50 Gy (range 30–50) in 5 consecutive fractions. Actually, only one patient had a prescription of 30 Gy, two patients had a prescription of 35 Gy, 3 patients had a prescription of 40 Gy, and 24 patients had a prescription of 50 Gy. The reason for a different level of dose prescription is related to the characteristics of the patients and the tumors treated. Indeed, our reference dose level was 50 Gy in 5 fractions. However, we delivered lower dose levels for 3 patients who did not have pancreatic adenocarcinoma. One patient with a primary pancreatic neuroendocrine tumor with a single liver metastasis was treated with 30 Gy in 5 fractions because of the tumor size and metastatic status. Two patients with oligometastatic renal clear cell carcinoma were treated with 35 Gy and 40 Gy in 5 fractions due to the oligometastatic status outside the pancreas. One patient with metastatic pancreatic adenocarcinoma was treated with 35 Gy in 5 fractions. Finally, two patients with borderline resectable pancreatic cancer (BRPC) in whom pancreatic surgery was considered after SMART received 40 Gy in 5 fractions. In the end, only one of these two patients underwent surgery. Treatment planning was done using the ViewRay^®^ Treatment Planning System (TPS), using a Monte Carlo algorithm, with normalization on D50 (100% of the prescribed dose covers 50% of the target volume), trying to ensure 95% PTVopt coverage within the 95% isodose and 99% GTV coverage with the 95% isodose. Treatment was delivered using step-and-shoot intensity-modulated radiation therapy (IMRT) with 6-MV photons and approximately 15–20 beams and 70–90 segments. No concomitant chemotherapy was administered during radiotherapy.

**Table 1:** Organ at risk dose constraints.

Organ	Dose constraints (5 fractions)
Esophagus	Dmax < 35 Gy V_19.5Gy_ < 5 cm^3^
Stomach	Dmax < 32 Gy V_18Gy_ < 10 cm^3^
Duodenum	Dmax < 32 Gy V_18Gy_ < 5 cm^3^
Small intestine	Dmax < 32 Gy V_19.5Gy_ < 5 cm^3^
Large intestine	Dmax < 32 Gy V_25Gy_ < 5 cm^3^
Liver	V_ < 15Gy_ > 700 cm^3^
Kidneys	V_ < 17.5Gy_ > 200 cm^3^ V_18Gy_ < 33% V_ < 14.5Gy_ > 130 cm^3^ (if single kidney)
Spinal cord	Dmax < 25 Gy
Heart	Dmax < 30 Gy V_24Gy_ < 15 cm^3^

### Daily Adaptive Treatment Workflow

After daily TRUFISP image acquisition, patients were positioned to the pancreatic area. After rigid registration of the GTV, OAR contours were propagated on the daily MR image using deformable image registration. OAR contours not considered optimal were modified by the physician (especially digestive OAR contours). The initial plan was then evaluated by the physician and the physicist. If all dose constraints were met, no adaptation was required (non-adapted fractions). If a decrease in tumor coverage and/or unacceptable OAR dose constraints were observed, the initial plan was optimized on the integrated TPS (adapted fractions). The electron density map (transferred from the CT to MR images) and the skin contour were checked to ensure correct dose recalculation (11). Quality assurance of the newly optimized plan was performed by recalculating the plan with a secondary Monte Carlo algorithm before irradiation. Tracking was ensured by following a structure with good spontaneous contrast on MRIdian acquisition (usually the GTV itself) on sagittal images obtained by cine-MR. The beam was turned off when more than 5% of the tracked structure was outside the threshold of 3 mm from its initial position.

### Clinical Assessment, Dosimetric Evaluation, and Endpoints

The primary endpoint was acute toxicities. Secondary endpoints were late toxicities; the impact of the adaptive treatment on the target volume coverage and OAR sparing; the LC rate defined by the Response Evaluation Criteria in Solid Tumors (RECIST) criteria including local complete response (CR), local partial response (PR), and local stable disease (SD); the distant metastasis-free survival (DMFS) based on clinical, radiological, and biological assessment; and OS.

Follow-up started on the first day of SMART treatment until the death or latest news for each patient. Acute toxicities were defined as toxicities occurring during treatment until 3 months posttreatment. Late toxicities were defined as toxicities occurring after 3 months posttreatment.

All patients were assessed after treatment at 1 month and then every 3 months. The assessment consisted of clinical, radiological (CT scan, MRI, or PET scan), and biological (including tumoral markers carcinoembryonic antigen (CEA) and CA 19.9) evaluations at each visit. All toxicity events were reported according to the Common Terminology Criteria for Adverse Events (CTCAE) v5.0 at each clinical examination.

For each adapted fraction delivery, the predicted plan (initial plan on the daily image) and the delivered plan (new plan on the daily image) were compared *a posteriori* with the initial plan. PTV and GTV coverage (D2%, D95%, D98%, V100%, V95%, and V90%) values as well as OAR maximum dose and volumetric doses were recorded.

### Statistical Analysis

For survival analysis, median follow-up was estimated using the Kaplan–Meier method. OS was defined as the time between the end of chemotherapy or SMART and death by any cause. Alive patients were censured at the date of the last follow-up. Progression-free survival (PFS) was defined as the time between the end of chemotherapy or SMART and local relapse, metastatic relapse, or death by any cause. DMFS was defined as the time between the end of chemotherapy or SMART and distant relapse or death by any cause. LC was defined as the absence of progression of the primary pancreatic tumor. A subgroup analysis of each of these parameters was realized for LAPC and BRPC and, among those, between resected and non-resected patients. Comparison of the survival curves between the resected patients and the non-resected patients was performed by the log-rank (Mantel–Cox) test with hazard ratio (HR) (Mantel–Haenszel) calculation.

For each adapted fraction delivery, the predicted plan (initial plan on the daily image) and the delivered plan (new plan on the daily image) were compared *a posteriori* by a paired Wilcoxon test. PTV and GTV coverage values as well as OAR maximum doses and volumetric doses were recorded. Statistical analyses were performed using Stata v16.0, RStudio, and GraphPad PRISM v9.

## Results

### Patient and Treatment Characteristics

Between October 2019 and April 2021, thirty consecutive patients treated with SMART for an unresectable pancreatic tumor were included in our prospective registry study. Median follow-up was 9.7 months (95% CI: 5.85–11.86) for the whole cohort and 10.64 months (95% CI: 5.85–11.86) for pancreatic adenocarcinoma patients. Patient and treatment characteristics are described in **Table 2**. The median age was 64.5 years (range 44–85). The proportion of men and women was well balanced. Borderline or locally advanced pancreatic adenocarcinomas represented 22 patients (77%). There were also 1 patient (3%) with resectable pancreatic adenocarcinoma but unfit for surgery, 3 patients (10%) with oligometastatic disease from pancreatic adenocarcinoma, 1 patient (3%) with pancreatic neuroendocrine tumor, and 2 patients (6%) with pancreatic metastasis from kidney tumors. Twenty-eight patients (94%) received chemotherapy before radiotherapy, mainly induction FOLFIRINOX regimen (73%) with a median of eight cycles (range 4–14). Four of these patients had to switch for FOLFOX, FOLFIRI, GEMCITABINE alone, or GEMCITABINE-ABRAXANE protocol because of tolerance issues. Serum CA 19.9 was initially available for 25 (83%) patients. The median value of serum CA 19.9 decreased from 321 (range, 6–1884) to 108 UI/ml (range, 6–802) between diagnosis and the start of SMART. Based on MRI or CT findings at diagnosis, 77% of patients were without nodal invasion. The average size of a pancreatic tumor before SMART was 31.5 mm (range 16–53). The tumor was mainly localized in the pancreas head (57%).

**Table 2:** Baseline characteristics.

**Sex**	
Women	15 (50%)
Men	15 (50%)
Median age (range)	64.5 years (44–85)
**Pathology**	
Pancreatic adenocarcinoma (PA)	27 (90%)
Pancreatic neuroendocrine tumor	1 (3.33%)
Metastasis from kidney tumor	2 (6.67%)
**Stage among PA**	
Resectable	1 (3.33%)
Borderline	3 (10%)
Locally advanced	19 (63.33%)
Local relapse	1 (3.33%)
Metastatic	3 (10%)
**Previous treatment**	
Chemotherapy	28 (93.33%)
Pancreatic surgery	3 (10%)
CAR-T cells	1 (3.33%)
None	1 (3.33%)
**ECOG score**	
0	11 (36.67%)
1	17 (56.67%)
2	2 (6.67%)
3	0 (0%)
**Chemotherapy regimen for PA**	
FOLFIRINOX	22 (73.33%)
GEMCITABINE-ABRAXANE	2 (6.67%)
FOLFOX	4 (13.33%)
GEMCITABINE	2 (6.67%)
FOLFIRI	1 (3.33%)
Several protocols*	4 (13.33%)
**Localization**	
Head	16 (57.17%)
Body/tail	12 (42.86%)
Unknown	2
**Lymph node involvement°**	
Yes	7 (23.33%)
No	23 (76.67%)
**Median CA 19.9 at diagnosis** (range)	321 UI/ml (6–1,884)
**Median CA 19.9 before SMART** (range)	108 UI/ml (6–802)
**Average size of pancreatic tumor** (range)	31.5 mm (16–53)

ECOG, Eastern Cooperative Oncology Group; SMART, stereotactic MR-guided adaptive radiotherapy.

*Because of tolerance issues with FOLFIRINOX.

°On CT/MRI/PET.

### Initial Treatment Plans

All patients underwent five daily consecutive fractions. The prescribed dose was 50 Gy for 24 patients, 40 Gy for 3 patients, 35 Gy for 2 patient, and 30 Gy for one patient. The median fraction duration was 86 min (range 64–133). The median PTV was 67.4 cm^3^ (range 6.9–138.7). **Table 3** presents the dosimetric data of initial plans.

**Table 3:** Median (min–max) dosimetric data for initial plans.

Total dose (Gy)	50 (24 patients) 40 (3 patients) 35 (2 patient) 30 (1 patient)
Total treatment duration (days)	6 (5 – 14)
Fraction dose (Gy)	10 (6 – 10)
Median PTV (cm^3^)	68.6 (6.9 – 138.7)
Fraction duration (min)	89.8 (64 – 133)
**PTVopt**	
V100% (%)	58.1 (40.5 – 83.1)
V95% (%)	90.6 (68.9 – 99.9)
V80% (%)	99.6 (92.9 – 100)
D98% (Gy)	41.9 (28.5 – 47.8)
D95% (Gy)	44.6 (29.1 – 48.6)
D2% (Gy)	53 (32.2 – 55.2)
**PTV**	
V100% (%)	50 (33.8 – 78.5)
V95% (%)	78.9 (57.5 – 98.5)
V80% (%)	90.9 (72.7 – 99.9)
D98% (Gy)	23.5 (12.6 – 84.4)
D95% (Gy)	29.3 (15.2 – 48.6)
D2% (Gy)	53 (32.1 – 55.1)
**GTV**	
V100% (%)	61.7 (40.5 – 93.3)
V95% (%)	89.8 (65.1 – 100)
V80% (%)	96.1 (77.9 – 100)
D98% (Gy)	32.9 (17.8 – 49.2)
D95% (Gy)	38.6 (22.3 – 49.6)
D2% (Gy)	53 (32.3 – 55.9)
**Kidney**	
V_18Gy_ (cm^3^)	2.5 (0 – 16.6)
**Spinal cord**	
Dmax (Gy)	18 (7.61 – 22.9)
**Stomach**	
Dmax (Gy)	29.5 (0.9 – 32.8)
V_18Gy_ (cm^3^)	9.8 (0 – 30.5)
**Duodenum**	
Dmax (Gy)	29.4 (20.4 – 33.4)
V_18Gy_ (cm^3^)	4.2 (0.9 – 11.4)
**Small intestine**	
Dmax (Gy)	27.6 (2.8 – 34)
V_19.5Gy_ (cm^3^)	3.1 (0 – 9.1)
**Large intestine**	
Dmax (Gy)	27.8 (5.9 – 32.9)
V_25Gy_ (cm^3^)	1.8 (0 – 2.5)

PTV, planning target volume; GTV, gross tumor volume; PTVopt, PTV optimized.

### Dosimetric Benefits of Adaptive Method

All fractions (150) were adapted because of a dosimetric benefit obtained either on PTV coverage or on OAR protection. Adaptation was performed because of stomach, duodenum, or jejunum Dmax violation on predicted plans, as follows:

- 2 out of 5 fractions (40%) for a prescription dose of 30 Gy
- 7 out of 10 fractions (70%) for a prescription dose of 35 Gy
- 15 out of 15 fractions (100%) for a prescription dose of 40 Gy
- 110 out of 120 fractions (91.67%) for a prescription dose of 50 Gy

The remaining fractions were adapted to improve target volume coverage.

**Figure 1:**
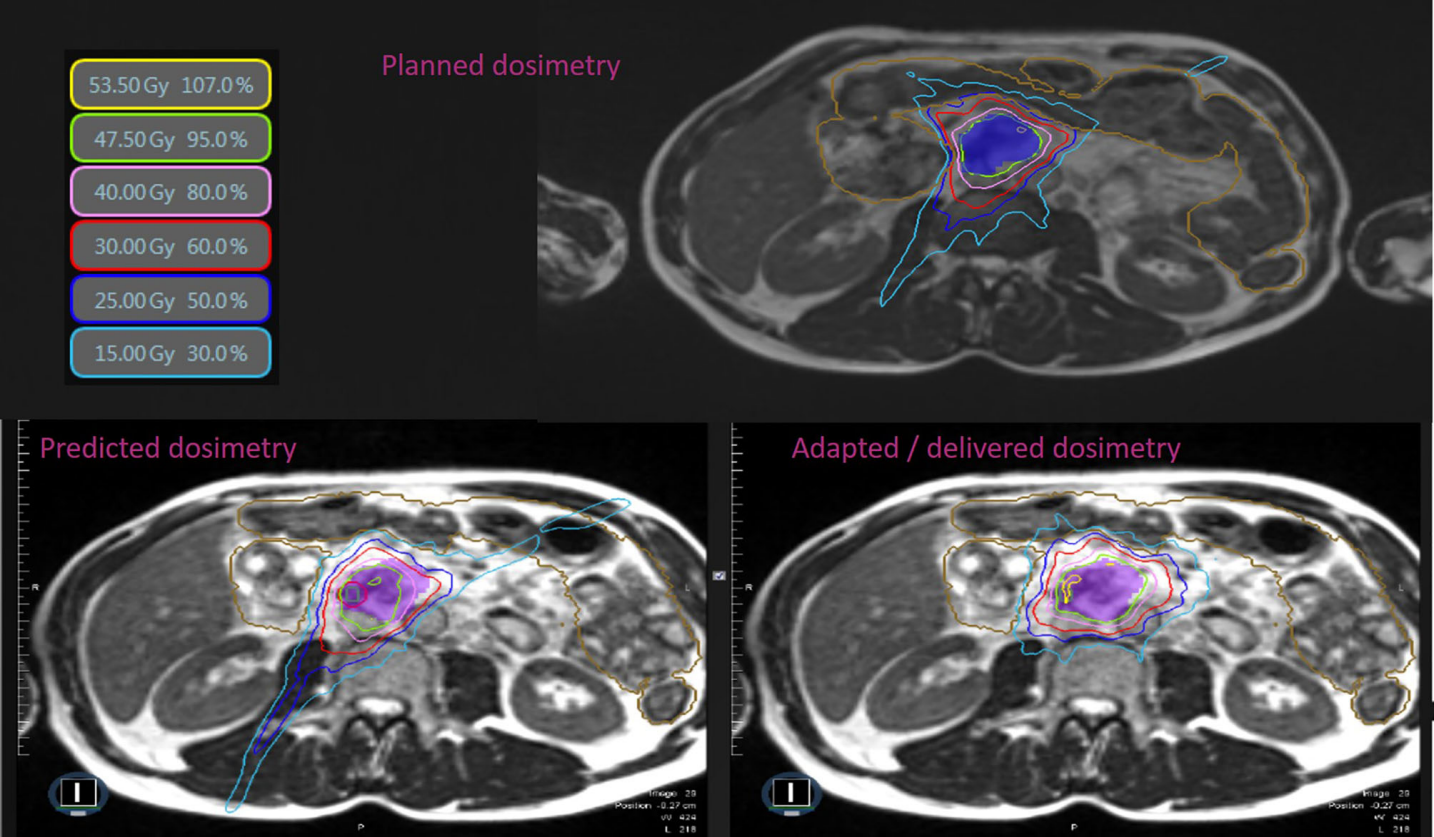
Typical SMART dosimetry showing planned, predicted, and adapted/delivered dosimetry. Comparison of dose distribution between planned, predicted, and adapted/delivered dosimetry on MR 0.35-T TRUFISP images for a prescription of 50 Gy in 5 fractions in LAPC. Isodose line 53.5 Gy in yellow, 47.5 Gy in green, 40 Gy in rose, 30 Gy in red, 25 Gy in blue, and 15 Gy in cyan. Small intestine in brown. LAPC, locally advanced pancreatic cancer; SMART, stereotactic MR-guided adaptive radiotherapy.

**Figure 2:**
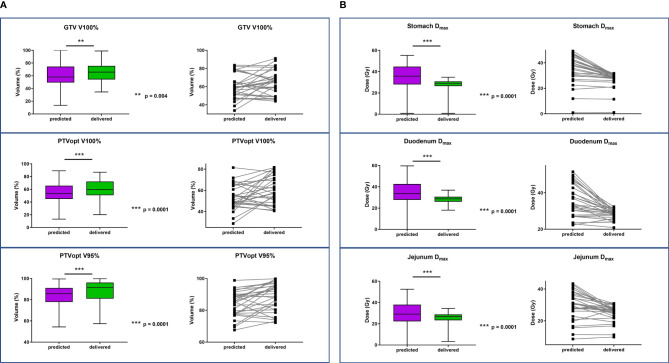
Dosimetric comparison between predicted and adapted plans. **(A)** Target volumes coverage (GTV V100%, PTVopt, V100% and PTVopt V95%). **(B)** OAR sparing (Dmax to stomach, duodenum, and jejunum). The three images on the left part of the figures represent the 150 predicted fractions vs. 150 adapted fractions (5 fractions for 30 patients). Whiskers show minimal to maximal values, and boxes show the mean values with standard deviations. The three images on the right part of the figures represent the variation for each patient (one line represents one patient) from the predicted values (left) to the adapted values (right). In order to limit the number of lines (30 lines) and to make the figure readable, we have calculated and plotted the average values of the 5 predicted plans (left) and the average values of the 5 adapted plans (right). GTV, gross tumor volume; PTVopt, planning target volume optimized; OAR, organ at risk.

The mean treatment duration of adapted fractions was 90 min, including patient preparation, positioning, image acquisition, image registration, OAR recontouring, plan adaptation, and treatment delivery. Average dosimetric data and comparison between predicted and adapted plans are available in **Table 4**. PTV coverage was significantly improved for adapted plans compared to predicted plans (mean PTV V95% increase of 2.2%, p < 0.01), as was the PTV optimized coverage (mean PTV V95% increase of 4.3%, p < 0.01). The adaptation of the plan also significantly improved dosimetric measures for OAR, except for the kidneys. **Figure 1** shows an example of the benefit of adaptation on PTV coverage for a given fraction. **Figure 2** shows a dosimetric comparison between predicted and adapted plans for target volumes coverage (GTV V100%, PTVopt V100%, and PTVopt V95%) (**Figure 2A**) and for OAR sparing (Dmax to the stomach, duodenum, and jejunum) (**Figure 2B**). The benefit of adaptive plans vs. predicted plans on target volumes is less obvious than on OARs because PTV coverage values on predicted plans are often in parallel with unacceptable OAR values (unacceptable plans that cannot be delivered to patients).

**Table 4:** Average target volume and OAR dosimetric results for predicted and adapted plans.

Target volume / OAR	Predicted plan [standard deviation]	Adapted plan [standard deviation]	p-Value
**PTVopt**			
V100%	53.7% [14.5%]	60.2% [14.4%]	**<0.01**
V95%	84% [9.1%]	88.3% [9.4%]	**<0.01**
V80%	95.4% [4.8%]	98.4% [2.2%]	**<0.01**
D98%	34.7 Gy [7.3 Gy]	40.1 Gy [5 Gy]	**<0.01**
D95%	38.9 Gy [6.4 Gy]	42.3 Gy [4.9 Gy]	**<0.01**
D2%	50.7 Gy [6.3 Gy]	50.6 Gy [6 Gy]	0.31
**PTV**			
V100%	47% [12.6%]	51.7% [13%]	**<0.01**
V95%	74.4% [10.6%]	76.6% [12%]	**<0.01**
V80%	87.9% [8.1%]	88.6% [8.2%]	0.22
D98%	25.4 Gy [8.5 Gy]	26.2 Gy [9.8 Gy]	0.5
D95%	30.4 Gy [8.8 Gy]	31.4 Gy [10.1 Gy]	0.35
D2%	50.7 Gy [6.3 Gy]	50.3 Gy [6.5 Gy]	0.2
**GTV**			
V100%	60% [15.9%]	65.1% [14.3%]	**<0.01**
V95%	85.9% [9.9%]	86.8% [10.4%]	0.15
V80%	93.7% [6.4%]	93.8% [6.8%]	0.72
D98%	32.7 Gy [8.9 Gy]	33 Gy [10.9 Gy]	0.93
D95%	37.2 Gy [8.4 Gy]	37.4 Gy [9.9 Gy]	0.65
D2%	50.9 Gy [6.3 Gy]	50.8 Gy [6 Gy]	0.60
**Kidney**			
V_18Gy_	4.2 cm^3^ [4.6%]	4.7 cm^3^ [5.1%]	**<0.01**
**Spinal cord**			
Dmax	17.2 Gy [3.7 Gy]	17.5 Gy [3.2 Gy]	0.25
**Stomach**			
V_18Gy_	15.2 cm^3^ [11.2 cm^3^]	12.1 cm^3^ [8.8 cm^3^]	**<0.01**
Dmax	35.2 Gy [11.8 Gy]	27.2 Gy [6.6 Gy]	**<0.01**
**Duodenum**			
V_18Gy_	6.6 cm^3^ [5.7 cm^3^]	4.5 cm^3^ [3 cm^3^]	**<0.01**
Dmax	35.4 Gy [10.1 Gy]	28.1 Gy [3.52 Gy]	**<0.01**
**Small intestine**			
V_19.5Gy_	3.8 cm^3^ [5.1 cm^3^]	2.4 cm^3^ [2.7 cm^3^]	**<0.01**
Dmax	29.5 Gy [10.9 Gy]	25 Gy [6.2 Gy]	**<0.01**
**Large intestine**			
V_25Gy_	1 cm^3^ [1.9 cm^3^]	0.4 cm^3^ [0.8 cm^3^]	**<0.01**
Dmax	24.7 Gy [10.3 Gy]	23.1 Gy [7.3 Gy]	**<0.01**

OAR, organ at risk; PTV, planning target volume; GTV, gross tumor volume; PTVopt, PTV optimized.

Bold values are statistically significant differences (p< 0.05).

### Toxicities

No patients presented grade > 2 acute toxicities, and 13 patients presented grade 1–2 acute toxicities (asthenia (grade 1: 40%), abdominal pain (grade 1: 40%), nausea/vomiting (grade 1: 23.3%, grade 2: 20%), and diarrhea (grade 1: 23.3%, grade 2: 3.3%).

After surgery, one patient presented a digestive fistula, and another one presented an abdominal aneurism, highly suggestive of immediate postoperative complications from head pancreatic surgery consecutive to the anastomosis and vascular reconstruction problems. Both underwent additional surgical procedures with complete resolution afterwards.

With a median follow-up of 9.7 months for the whole cohort (95% CI: 5.85–11.86), no grade > 2 late toxicities were observed. Toxicities between resected and non-resected patients were not significantly different. More details are available in **Table 5**.

**Table 5:** SMART-related acute and late toxicities.

CTCAE v5.0	Acute toxicity (0–90 days)	Late toxicity (90 days–1 year)
Abdominal pain		
g0	18 (60%)	13 (43.3%)
g1	12 (40%)	8 (26.7%)
g2	0	1 (3.3%)
g3	0	0
Ongoing	0	7 (23.3%)
Nausea/Vomiting		
g0	17 (56.7%)	19 (63.3%)
g1	7 (23.3%)	2 (6.7%)
g2	6 (20%)	2 (6.7%)
g3	0	0
Ongoing	0	7 (23.3%)
Gastritis/enteritis		
g0	29 (96.7%)	23 (66.7%)
g1	1 (3.3%)	0
g2	0	0
g3	0	0
Ongoing	0	7 (23.3%)
Gastroduodenal ulcer		
g0	30 (100%)	23 (66.7%)
g1	0	0
g2	0	0
g3	0	0
Ongoing	0	7 (23.3%)
Digestive fistula		
g0	30 (100%)	23 (66.7%)
g1	0	0
g2	0	0
g3	0	0
Ongoing	0	7 (23.3%)
Diarrhea		
g0	22 (63.3%)	16 (53.3%)
g1	7 (23.3%)	4 (13.3%)
g2	1 (3.3%)	3 (10%)
g3	0	0
Ongoing	0	7 (23.3%)

SMART, stereotactic MR-guided adaptive radiotherapy; CTCAE, Common Terminology Criteria for Adverse Events.

### Survival Analysis

#### Whole Cohort

**Figure 3:**
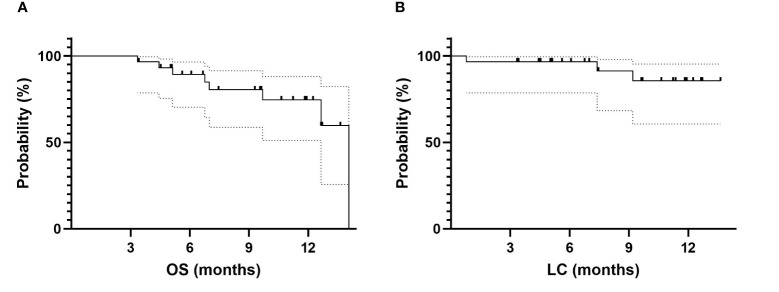
Survival date for the whole cohort. **(A)** OS for the whole cohort. **(B)** LC for the whole cohort. OS, overall survival; LC, local control.

The median OS was 14.1 months. The 6-month OS from SMART completion was 89% (95% CI: 70%–96%). The 1-year OS from SMART completion was 75% (95% CI: 51%–88%) (**Figure 3A**).

LC at 6 months and 1 year was respectively 97% (95% CI: 79–99.5%) and 86% (95% CI: 61%–95%) (**Figure 3B**). Among 3 local relapses (10%), 2 were located on the field edge and 1 inside the field.

#### Locally Advanced Pancreatic Cancer and Borderline Resectable Pancreatic Cancer Patients

LAPC and BRPC patients had a median follow-up of 10.64 months (95% CI: 5.85–11.86) from SMART.

**Figure 4:**
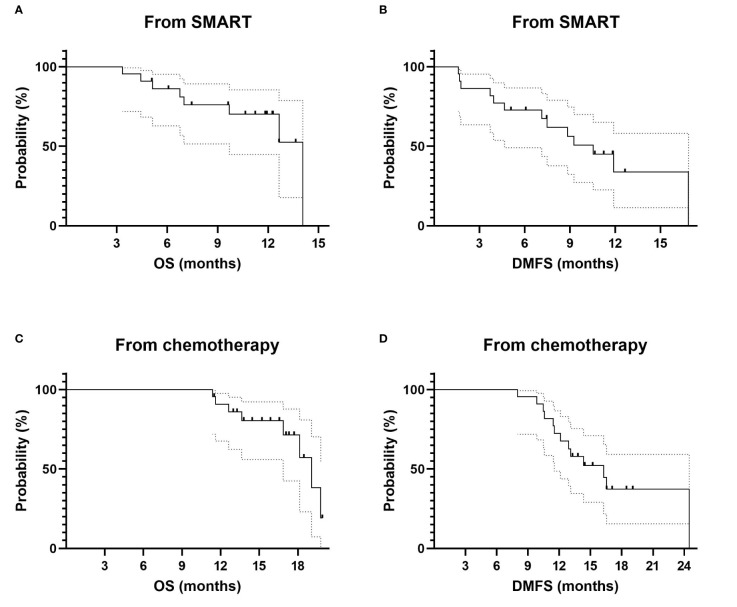
Survival data for LAPC and BRPC patients: comparison between resected and unresected patients. **(A)** OS from SMART completion. **(B)** DMFS from SMART completion. **(C)** OS from chemotherapy start. **(D)** DMFS from chemotherapy start. LAPC, locally advanced pancreatic cancer; BRPC, borderline resectable pancreatic cancer; OS, overall survival; SMART, stereotactic MR-guided adaptive radiotherapy; DMFS, distant metastasis-free survival.

The median OS was 14.1 months. The 6-month OS from SMART completion was 76% (95% CI: 51%–89%). The 1-year OS from SMART completion was 70% (95% CI: 45%–85%) (**Figure 4A**). The median DMFS from SMART completion was 10.5 months. The 6-month DMFS from SMART completion was 73% (95% CI: 49%–87%). The 1-year DMFS from SMART completion was 34% (95% CI: 11%–58%) (**Figure 4B**).

The median OS and 1-year OS from initiation of induction chemotherapy were 19.1 months and 91% (95% CI: (68%–98%), respectively (**Figure 4C**).

The median DMFS and 1-year DMFS from initiation of induction chemotherapy were 16.3 months and 72% (95% CI: 49%–87%), respectively (**Figure 4D**).

The median serum CA 19.9 initially decreased with a nadir at 6 months (70 UI/ml range, 1.1–692) and increased at 1 year (147 UI/ml range, 9–792).

**Figure 5:**
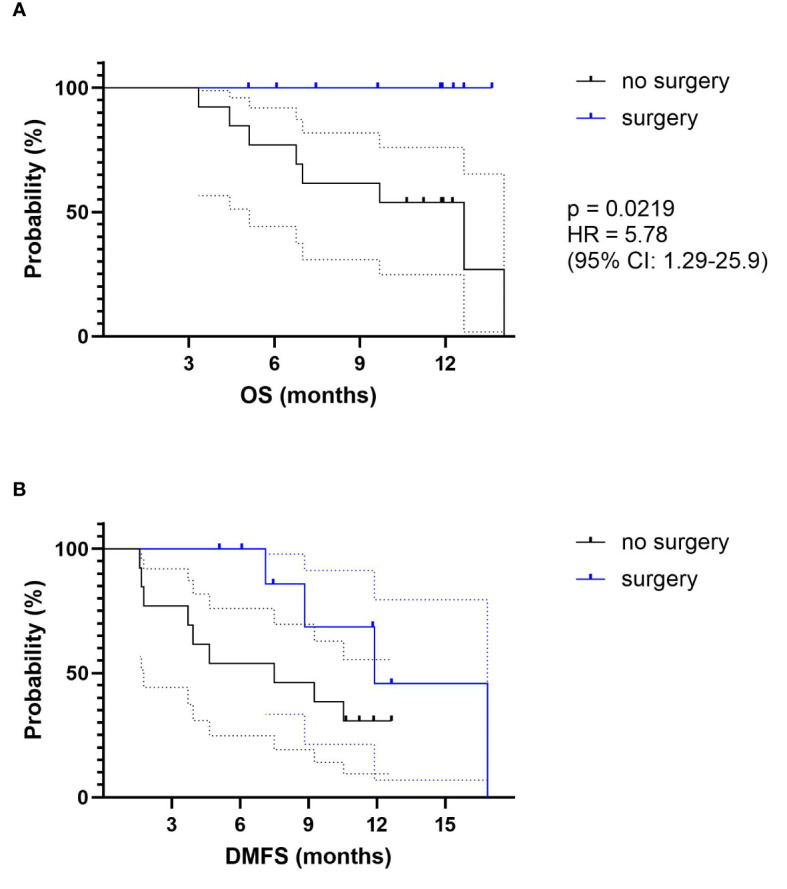
Survival data for LAPC and BRPC patients: comparison between resected (in blue) and unresected patients (in black). **(A)** OS from SMART completion. **(B)** DMFS from SMART completion. OS, overall survival, DMFS, distant metastasis-free survival.

Primary adenocarcinoma patients considered with a responsive disease (CA 19.9 decrease and radiological assessment classified as stable, or in response according to RECIST 1.1 classification) and clinically fit were proposed for pancreatic surgery. For the selected patient, after agreement of the multidisciplinary staff, including trained surgeons, duodeno-pancreatectomy or spleno-pancreatectomy was realized, depending on initial tumor location. Consequently, nine patients (8 out of 19 patients (42.1%) with initial LAPC and one out of 3 patients (33.3%) with BRPC) were resected. Histologically, the average pathologic therapeutic effect was evaluated at 64% (range 10%–95%), mainly classified ypT2N0 (56%). There was no CR. All patients underwent complete surgery with negative margins (R0). Among them, 3 patients had a metastatic relapse, and one of them had also a local relapse on the field boundary. To date, all resected patients are still alive. Resected patients had a significantly better OS as compared to unresected patients (p = 0.0219, HR = 5.78 (95% CI: 1.29–25.9) (**Figure 5A**). DMFS was not significantly different between resected and unresected patients (**Figure 5B**).

## Discussion

Management of pancreatic tumors remains a major challenge for surgeons, radiation oncologists, and medical oncologists due to the anatomical location of the pancreas in contact with vascular and digestive structures and because of the poor prognosis of pancreatic adenocarcinoma. Until now, surgical resection is the only curative modality of pancreatic adenocarcinoma, and the publication of adjuvant FOLFIRINOX results for resected patients has dramatically improved OS in this population with a median OS of 54.4 months (12). Unfortunately, only 10% of patients are resectable at diagnosis; 10% are considered borderline resectable and 30% unresectable or locally advanced (2). Unresected patients have a poor prognosis, with median OS of approximately 11 months (13). In a recent study on LAPC, the 3-year OS was 43% in the resected group vs. 6.5% in the unresected group (14). However, the better prognosis of resected patients may also reflect a better response to neoadjuvant treatment, since only good responders will ultimately undergo surgery. Thus, it seems important to try to improve induction treatments in order to increase the therapeutic response and make more patients resectable.

Radiotherapy remains a controversial treatment in LAPC since the LAP07 trial failed to prove a survival benefit in patients receiving fractionated radiochemotherapy after induction chemotherapy. Nevertheless, the reference protocols for induction chemotherapy, but especially the available radiotherapy techniques, have evolved considerably as compared to the treatments used in this clinical trial. SBRT uses an advanced technological approach to improve nearby OAR sparing while ensuring a correct coverage of the target volumes. Moreover, this treatment is delivered in a limited number of fractions, which improves the comfort and quality of life of patients. Finally, SBRT appears to be an attractive modality for the treatment of this radioresistant tumor type (15). A recent meta-analysis comparing SBRT and radiochemotherapy with conventional fractionation suggested a benefit in favor of SBRT, with 2-year OS of 26.9% vs. 13.7%. In addition, this study demonstrated also a benefit in terms of tolerance, with 5.6% acute grade 3/4 toxicities versus 37.7%, without differences of late grade 3/4 toxicities (16). Looking individually at the prospective studies evaluating SBRT in LAPC, the prescription dose varied from 15 Gy in 1 fraction to 45 Gy in 6 fractions (17–21). The proximity of digestive organs was the main issue of these studies with the rate of severe (grade > 2) gastrointestinal (GI) toxicities ranging from 5% up to 22%, especially for treatment in one fraction (19, 22–25). All of these trials used Linac or CyberKnife, with no possibility of daily adaptation, probably partially explaining the rate of GI toxicities (5, 6).

For this reason, adaptive radiotherapy seems to be a good solution to improve digestive organs sparing while keeping a high prescription dose, by daily adaptation of dosimetric plan to daily anatomy. With the development of MR-guided radiotherapy, new possibilities are offered for the treatment of LAPC. SMART is a technique allowing high prescription doses by 1) using good soft-tissue contrast of MRI for a precise delineation of target volumes and OAR, 2) using integrated TPS for daily adaptation of dosimetry, and 3) tracking of target volume using continuous cine-MR acquisitions (7, 8, 26).

Two series of SMART for the treatment of LAPC recently reported very encouraging results. The prescribed dose was 40 Gy (one patient), 45 Gy (4 patients), and 50 Gy (30 patients) in 5 fractions in the first study with a median follow-up of 10.3 months from SMART completion (27). In the second study, the prescribed dose was 50 Gy in 5 fractions for all patients with a median follow-up of 16 months from diagnosis and not SMART completion. The tolerance was excellent with only one (3%) acute grade 3 toxicity and one (3%) late grade 3 toxicity in the first study (27) and no late grade 3 toxicity and only 2 (4.6%) acute grade 3 toxicities in the second one (28). There were no grade > 3 toxicities in both studies. Our results are in accordance with their results, as we did not report any grade > 2 toxicities. The most frequent acute toxicities were asthenia (40%), abdominal pain (40%), and nausea/vomiting (43%), with no need for treatment interruption. Late tolerance was excellent too, with only grade 1 toxicities. Two patients had postoperative complications that resolved and can be considered unrelated to radiotherapy.

The benefits of adaptive treatment have already been demonstrated in other studies using SMART for different clinical indications. In lung tumors, the average gain per fraction for the PTV coverage (V100%) was 4.4% (29). For prostate reirradiation, we showed a benefit of adaptation on PTV coverage, without exceeding doses to OAR (30). In another study of different tumor localizations treated with SMART, 35/61 fractions were adapted because of OAR violation and led to better PTV coverage (7). In our study, we chose to prioritize OAR dose constraints. All patients had a daily adaptive treatment, usually for OAR dose constraints violation. Similarly, our adapted plans showed a significantly better PTV and PTV optimized coverage (an increase of mean PTV V95% of 2.2% and 4.3% respectively, p < 0.01). There was also a significant benefit of adaptation on stomach, duodenum, and small and large intestine dose constraints. In our experience, adaptive radiotherapy seems to be compulsory for the treatment of abdominal targets at this dose level (>40 Gy in 5 fractions).

In our study, the median OS calculated from SMART was 14.1 months in both the whole cohort and the BRPC and LAPC cohorts. In the other SMART series for LAPC and BRPC with the same dose prescription (50 Gy in 5 fractions), the median OS was 9.8 and 15.7 months (27, 28). The 1-year LC in our cohort was 86%, similar to respectively 87% and 84.3% in the studies of Chuong *et al.* and Hassanzadeh *et al.*, confirming that SMART achieves a high LC rate in LAPC. LC is of particular importance for LAPC patients, as local progression is a frequent cause of morbidity and mortality (31). Indeed, Rudra *et al.* showed that increasing the BED_10_ over 70 Gy translated into OS benefit (2-year OS of 49% when BED_10_ > 70 Gy vs. 30% when BED_10_ < 70 Gy) (10). This suggests that LC plays a role in OS too. A recent review demonstrated the dose–response effect using SBRT for pancreatic cancers, from 70% 1-year LC for equivalent 24 Gy in 3 fractions to 86% for equivalent 30 to 36 Gy in 3 fractions, confirming the necessity to prescribe high doses in this population (32). This is also suggested by the results of other retrospective studies using non-MR Linac and non-adaptive techniques, where 1-year LC ranged from 48.5% to 78% for prescription of 1 fraction of 24 Gy to 5 fractions of 6.6 Gy (33–36).

However, the median DMFS and 1-year DMFS from initiation of induction chemotherapy in our study were 16.3 months and 72%, but only 10.5 months and 34% from SMART, showing the frequent and quick metastatic dissemination of these cancers.

Our study is the first to report a high rate of secondary resection after SMART. Indeed, nine patients (8 out of 19 patients (42.1%) with initial LAPC and one out of 3 patients (33.3%) with BRPC) were resected. In the studies published by the Washington University and Miami teams on SMART for pancreatic cancers, the resection rate was respectively 9% (28) and 14% (27). All our patients had an R0 resection, and the average pathologic therapeutic effect was 64%. Resected patients in our study had a significant increase in OS (HR = 5.78 (95% CI: 1.29–25.9); p = 0.0219). We report the feasibility of pancreatic surgery after SMART, provided that these high-risk surgeries are carried out by trained surgical teams with significant experience in these procedures. These results lead us to pursue our aggressive strategy in this situation, especially as some lesions that appeared inoperable on the post-SMART scan were finally able to benefit from an R0 resection and a probable therapeutic benefit. Indeed, we confirmed the imaging struggles to assess resectability after neoadjuvant treatment.

Our study presents some limits. First, the number of patients is limited and the study is monocentric, but we must consider that SMART is a new technique available in a few centers. Second, our study population is heterogeneous, with three patients presenting a neuroendocrine tumor or pancreatic metastases of another primary. We decided to keep these patients for dosimetric and toxicity analysis, as the treatment site and anatomical and dosimetric characteristics were similar, but a subgroup analysis on BRPCs and LAPCs regarding survival data had to be performed. Then, our follow-up is limited, and our results need to be confirmed with a longer follow-up.

In our study, OS from SMART completion was 14.1 months, highlighting the poor prognosis of this patient population, despite a good LC rate (86% at 1 year). This result highlights the need for intensification of therapy and personalization of treatment according to the characteristics of the disease. We believe that the use of radiomics could play a part in this therapeutic personalization. Cusumano et al. used a delta radiomics approach for patients treated with SMART for pancreatic cancer. They identified a feature capable to predict 1-year LC with an AUC of 0.78 (37). Patients with a poor prognosis may be offered intensified systemic therapy or dose-escalated radiation therapy.

The first published results of SMART in the treatment of pancreatic tumors seem encouraging, and our clinical results in a prospective registry confirm the safety data and seem to show therapeutic benefit for patients. However, we need more prospective, multicenter data to confirm these trends. The first encouraging results of the multicenter SMART pancreas study sponsored by ViewRay were presented at ASTRO 2021 and seem to confirm the interest in the technique. In France, the GABRINOX ART trial is ongoing (38). This trial is evaluating an intensified and sequential chemotherapy regimen (Gabrinox) comprising Gembrax (Gemcitabine-Abraxane) and Folfirinox (5FU, oxaliplatin, and irinotecan) in patients with LAPC, followed by SMART in non-progressive patients after induction chemotherapy. In the United States, a trial is evaluating a combination of SMART and concomitant chemotherapy by gemcitabine or capecitabine (39). We hope that the results of these trials will give us robust results confirming the benefit of this technique for patients with pancreatic tumors.

## Conclusion

SMART for pancreatic tumors is feasible without limiting toxicities. Daily adaptation demonstrated a benefit for tumor coverage and OAR sparing. Acute and late toxicities were low. OS and LC rates were promising. SMART achieved a high secondary resection rate in LAPC patients. Surgery after SMART seemed to be feasible and might increase OS in these patients.

## Data Availability Statement

The raw data supporting the conclusions of this article will be made available by the authors, without undue reservation.

## Ethics Statement

The studies involving human participants were reviewed and approved by COMERE ICM. The patients/participants provided their written informed consent to participate in this study.

## Authors Contributions

Conceptualization: OR, KB, and MM. Methodology: SG and OR. Investigation: KB, MM, and OR. Supervision: OR. Writing—original draft preparation: OR, MM, and KB. Writing—review and editing: OR, MM, KB, MC, SG, EA, FP, MD, SV, PD, SS, MY, NA, PF, DA, RD, PEC, FRS, and SC. All authors have read and agreed to the published version of the manuscript.

## Conflict of Interest

The authors declare that the research was conducted in the absence of any commercial or financial relationships that could be construed as a potential conflict of interest.

## Publisher’s Note

All claims expressed in this article are solely those of the authors and do not necessarily represent those of their affiliated organizations, or those of the publisher, the editors and the reviewers. Any product that may be evaluated in this article, or claim that may be made by its manufacturer, is not guaranteed or endorsed by the publisher.
